# Image Statistics and the Representation of Material Properties in the Visual Cortex

**DOI:** 10.3389/fpsyg.2016.01185

**Published:** 2016-08-17

**Authors:** Elisabeth Baumgartner, Karl R. Gegenfurtner

**Affiliations:** Abteilung Allgemeine Psychologie, Fachbereich 06 Psychologie und Sportwissenschaft, Justus-Liebig-Universität GiessenGiessen, Germany

**Keywords:** fMRI, material perception, material properties, image statistics, MVPA

## Abstract

We explored perceived material properties (roughness, texturedness, and hardness) with a novel approach that compares perception, image statistics and brain activation, as measured with fMRI. We initially asked participants to rate 84 material images with respect to the above mentioned properties, and then scanned 15 of the participants with fMRI while they viewed the material images. The images were analyzed with a set of image statistics capturing their spatial frequency and texture properties. Linear classifiers were then applied to the image statistics as well as the voxel patterns of visually responsive voxels and early visual areas to discriminate between images with high and low perceptual ratings. Roughness and texturedness could be classified above chance level based on image statistics. Roughness and texturedness could also be classified based on the brain activation patterns in visual cortex, whereas hardness could not. Importantly, the agreement in classification based on image statistics and brain activation was also above chance level. Our results show that information about visual material properties is to a large degree contained in low-level image statistics, and that these image statistics are also partially reflected in brain activity patterns induced by the perception of material images.

## Introduction

The perception of material and surface properties is crucial for many aspects of our interaction with the environment, yet until now we have only a limited understanding of how this is achieved. For example, it is not yet well understood how the brain can quickly and successfully differentiate between a smooth, slippery object and a rough one that will provide a good grip. Since the neuronal processes underlying such judgments are largely unknown, we chose to investigate the question, how material properties are represented in the brain’s visual system. In particular, we examined what image features the brain might rely on during the processing of material properties and where in the brain information about material properties can be decoded.

In psychophysical research, material perception has received increasing attention over the past years. It has been pointed out that the visual system probably relies on sets of invariant image statistics, or cues, in order to estimate object and material properties, instead of carrying out costly computations to work out the physics of a visual scene (Nishida and Shinya, 1998; Motoyoshi et al., 2007; Fleming et al., 2011; Marlow et al., 2011, 2012; Giesel and Zaidi, 2013; for a review, see Fleming, 2014). For example, spatial frequency information (Giesel and Zaidi, 2013) or skewness of the luminance histogram (Motoyoshi et al., 2007) have been shown to influence material property perception. However, it is unclear how these image statistics act on a neuronal basis. Previous fMRI studies on texture and material perception found that tasks associated with visual material perception, like texture discrimination or material categorization, led to an increase in activation in medial regions of the ventral extrastriate cortex in human observers (Newman et al., 2005; Cant and Goodale, 2007, 2011; Cant et al., 2009; Jacobs et al., 2014). There has been very little work on the neural processing of individual material properties except gloss (monkeys: Nishio et al., 2012, humans: Sun et al., 2015). Very recently, Sun et al., 2016) showed that visual stimuli with different surface properties (e.g., rough vs. glossy) led to differential activity both in somatosensory cortex and in early visual areas.

Hiramatsu et al. (2011) conducted an fMRI experiment to investigate the categorical representation of visually presented materials in the human brain. By means of multivoxel pattern analysis, they were able to show that throughout the ventral stream, information about material categories can be decoded. Importantly, they could show that the representation of materials in early areas is strongly based on low-level image statistics like contrast, spatial frequency and color information. In higher visual areas, the representation of materials was observed to be based on perceptual similarity instead, i.e., it reflected participants’ judgments of material properties. These results were recently replicated in macaques (Goda et al., 2014). Even though category information is already present in V1, they conclude that a semantic or categorical distinction between materials does not arise before the fusiform gyrus/collateral sulcus. Several studies have demonstrated that perceptual representations of materials are arranged in a meaningful and consistent fashion (Rao and Lohse, 1996; Baumgartner et al., 2013; Fleming et al., 2013). Fleming et al. (2013), for example, showed that visual material properties are well defined and closely associated to category memebership, and Baumgartner et al. (2013) could show that visual and haptic material perception is robust and closely related.

While Hiramatsu et al.’s (2011) study convincingly demonstrated the existence of different representational levels in material perception, it remains unclear how the perception of different material properties arises and propagates in the human visual cortex, and what information these perceptions are based on. We were interested in where and how early in the visual system material properties are represented, and, importantly, which image statistics are used by the visual system during the perception of materials. We conducted this study to answer these fundamental questions about material property perception. Our goal was to relate perceived material properties of the images, their statistical properties, and the brain activation elicited by them. These three are intrinsically connected to each other, so we applied well-established methods that have been used to approach several similar problems in vision science (Britten et al., 1996; Stone and Krauzlis, 2003) in order to investigate how they relate to each other.

## Materials and Methods

### Participants

Fifteen participants completed the material property experiment, 11 of them in addition completed the retinotopy scans. However, only for 9 out of these 11 participants could we obtain reliable maps. Nine of our participants were female, six male. Mean age was 24.1 years. All were students at Giessen University and right-handed according to the Edinburgh Handedness Inventory (Oldfield, 1971). The study was approved by the local ethics committee and all participants signed a consent form according to the Declaration of Helsinki.

### Stimuli

**FIGURE 1:**
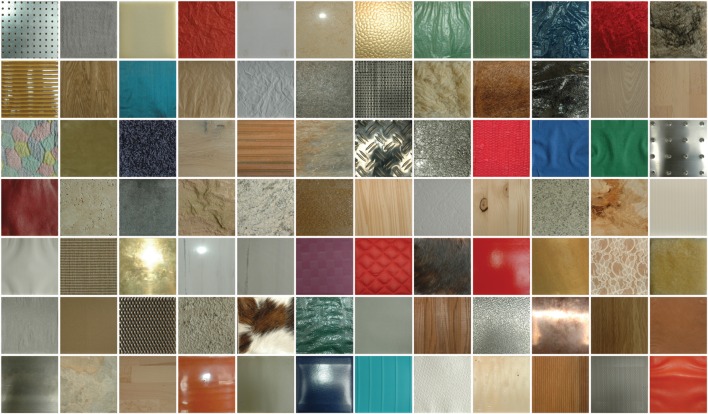
**The material photographs used in the fMRI experiment**.

We used a set of 84 pictures of material surfaces as stimuli in our experiment. These material surfaces had been collected for psychophysical studies on visual and haptic material perception in blind and sighted observers (Baumgartner et al., 2013, 2015). The actual samples were collected from different sources, mounted on tiles and photographed with a Nikon D70 SLR (Nikon, Tokyo, Japan). The stimuli were 14 cm × 14 cm flat patches of different samples of the following material categories: plastic, paper, fabric, leather, fur, stone, metal, and wood. For further information we refer to Baumgartner et al. (2013), where the stimulus database is described in great detail. We photographed the stimuli in the conditions of the experimental setup of our previous study from the participant’s point of view, with a window behind the participant/photographer and a point light source above the setup. Part of the photographs was also used in a study on image statistics by Wiebel et al. (2015). The photographs were then cropped to a size of 768 × 768 pixels such that only the material surface was retained (see **Figure 1**). Background luminance, when stimuli were presented on the projector in the experimental setup, was 176 cd/m^2^, mean stimulus luminance was 189 cd/m^2^. The images are made available for downloading^1^

### Rating and Material Properties

#### Procedure

We asked 6 of our 15 participants to indicate their assessments of the three material properties roughness, texturedness, and hardness on a 7-point Likert scale for each stimulus. In contrast to shape perception, material perception is intrinsically multimodal, so we wanted to use a visual, a haptic, and a bimodal material property. Baumgartner et al. (2013) found visual and haptic representations to be tightly linked. This is likely to be the result of learning processes (Goda et al., 2016). Therefore it is difficult to attribute certain properties to one of the senses but from our earlier work it seems that texturedness can be judged more reliably in the visual sense, while roughness is a property that is easily and reliably accessible to both senses from an object’s material surface. Hardness, in contrast, even though it can be derived to some degree using visual information (Baumgartner et al., 2013), is mainly perceived via the haptic sense and therefore served as a counterpart to the visually accessible properties. The photographs were presented on a computer screen in fully randomized order. Participants would rate each property one at a time, i.e., they rated one property for all stimuli, had a little break, and then rated the next property for all stimuli. The order of properties was also randomized. Participants were allowed to look at each stimulus as long as they wanted to. At the beginning of each property block, the participant was given a written definition of the property:

##### Roughness

How rough or smooth does the material seem to you? Low values indicate that the surface feels smooth; high values indicate that it feels rough.

##### Texturedness

How textured/patterned or homogeneous/uniform is the material’s surface? Low values indicate that the surface is uniform, high values indicate that the material has a pattern or texture.

##### Hardness

How hard or soft does the material seem to you? How much force would be required to change the shape of the material? Low values indicate that the surface feels soft; little force is required to change the shape of the material. High values indicate that it feels hard and cannot easily be deformed.

### Image Groups

For each material property, we formed groups of images with high and low ratings. We did this by ordering the images according to the ratings and then choosing the 25% percent of images with the highest property ratings for the high-ratings group, and the 25% percent of images with the lowest property ratings for the low-ratings group (21 images in each group). This resulted in two groups of images per material property with relatively low vs. relatively high values of the respective property, e.g., a group of rough images vs. a group of smooth images (for roughness).

### Image Statistics

In order to capture information about our material images we analyzed them according to the image statistics by Simoncelli and Portilla (1998) and Portilla and Simoncelli (1999, 2000). Although the algorithm has been extended to work on color images^2^, we worked with the initial version that uses grayscale images to keep the number of image statistics at a manageable level. The images were converted to luminance by multiplying R, G, and B with the relative luminance of the display device’s respective channel. The texture model by Portilla and Simoncelli is a complex model developed primarily for texture synthesis. The algorithm first extracts a large set of image statistics from the photographs. These image statistics are later used for an iterative image synthesis procedure. Here, however, we used the parameters computed by the model to describe our material images. We analyzed three types of statistics that the algorithm provides separately.

#### Pixel Statistics

Initially, the texture analysis algorithm computes marginal statistics of the textures gray level distribution (the number of pixels per gray level). These statistics are mean, variance, skew, kurtosis and the range (minimum and maximum) of the distribution. These statistics we will subsequently call ‘pixel statistics.’

#### Filter Statistics

The algorithm then decomposes an image into oriented, linear filters at different scales by means of a steerable pyramid (Simoncelli et al., 1992) and computes statistics to describe these filter outputs as well as relationships between them. Even though the model is inspired by filter responses in V1, the parameters do not necessarily correspond to particular statistics of V1 and V2 neurons’ responses. Non-spatial summary statistics of these responses that are implemented in the model might be computed at a later stage, presumably in V2 (Freeman and Simoncelli, 2011; Freeman et al., 2013).

In our case, we used a steerable pyramid with four scales and four orientations. First, the algorithm computes the local autocorrelation of the lowpass images that the steerable pyramid computes at each level. These autocorrelations capture regularity (periodic features) of the textures and salient spatial frequencies. Second, joint statistics of subband magnitude coefficients are computed. Specifically, correlations of neighbors in space, orientation and scale are used at this stage. These capture structures (e.g., edges) in images as well as ‘second order textures.’ Third, the algorithm computes cross-scale phase statistics. Hereby, the local relative phase between a subband’s coefficients and their neighbors at the next coarser scale is computed. These coefficients capture gradients within the textures, and can differentiate between edges and lines. In our case, the image analysis resulted in 877 image parameters.

#### Spatial Frequency Parameters

Spatial frequency has previously been described to directly affect perceived material properties (Giesel and Zaidi, 2013). The steerable pyramid of the model by Portilla and Simoncelli already captures the spatial frequency content to a certain extent. However, in order to exhaustively explore the spatial frequency content of our images, we reran the steerable pyramid with more filters, i.e., we built another steerable pyramid with twelve orientations and six scales. The 74 coefficients (12 × 6 + highpass and lowpass residuals) that resulted from the decomposition with the steerable pyramid constitute our third group of image statistics.

### FMRI Experiment

We showed each participant the 84 stimuli in randomized order. Between trials (i.e., images), we had intervals of at least 14 s (plus 0–2.5 s jitter) in order to keep the BOLD contamination of temporally neighboring images at a minimal level. Each stimulus was presented for 5 s. After 25% percent of trials, the participant was asked a rating question. The participant was asked to assess one of the three material properties for the stimulus he or she had just seen. The trials after which a rating question was presented were randomized such that it was impossible for the participants to predict if they would have to answer a question afterward while they looked at each material image. For each rating, we randomly chose a material property that we asked the participant to assess in order to avoid that participants had a certain material property in mind when looking at the stimuli. We did this to ensure that participants paid close attention to each stimulus. Participants had to assess the stimulus property on a 3-point Likert scale. They indicated their answers by pressing one of three response buttons. The experimental data was collected in a single functional run that lasted approximately 35 min. Please note that each material image was only seen once by each participant.

#### Stimulus Presentation

Stimuli were projected with an XGA-Projector (Epson, Model 7250, resolution: 1024 × 768) projection screen (460 × 350 mm) behind the scanner bore. The visual stimulation could be seen by means of a double mirror attached to the head coil (visual field 18° in horizontal and 16° in vertical, rectangular aperture). Our stimuli encompassed approximately 14° × 14° of the screen. We used Presentation software (Version 16, Neurobehavioral Systems TM, Albany, CA, USA) for stimulus presentation and response registration.

#### Scanning Parameters

Data was collected with a SIEMENS Symphony 1.5 Tesla MR imaging system with a quantum gradients system. The anatomical scan was collected in 160 T1-weighted sagittal images by means of a MP-RAGE sequence. Slice thickness was 1 mm. Afterward, a field map scan was acquired to measure inhomogeneities of the magnetic field. The functional data was collected using a single shot T2^∗^-weighted gradient-echo planar imaging (EPI) sequence, with 25 slices covering the whole brain, acquired in descending order (slice thickness 5 mm; 1 mm gap; *TA* = 2.4 s; *TR* = 2.5 s; *TE* = 55 ms; flip angle 90°; field of view 192 mm × 192 mm; matrix size 64 × 64; voxel size 3 mm × 3 mm × 5 mm.).

#### Preprocessing and Data Analysis

DICOM-files were converted to NIFTI-files using MRI Convert (Version 2.0; Lewis Center for NeuroImaging, Oregon). SPM8 (Statistical Parametric Mapping; Wellcome Department of Cognitive Neurology, London, UK) was used for pre-processing of the data. Pre-processing consisted of unwarping, realignment, co-registration, and smoothing (6 mm FWHM). Before the searchlight analysis procedure, anatomical and functional data were additionally normalized to the MNI template brain.

#### Retinotopy

Retinotopy stimuli consisted of a rotating wedge and an expanding circle with a high contrast (black and white) checkerboard pattern that changed phase with a frequency of 4 Hertz. Both wedge and circle were presented simultaneously, whereby the wedge stimulus completed 5 cyles per run (80 s/run) and the ring completed eight cycles per run (50 s/run). Participants had to fixate a gray fixation dot in the center of the screen and were asked to press a button whenever they noticed a color change of the fixation dot. Participants completed 3 or 4 runs of retinotopy.

Retinotopy data was collected in a separate (second) session. Stimulus presentation and scanning parameters were identical to those of the main experiment. Retinotopy data was unwarped, co-registered, and smoothed (6 mm FWHM). We delineated retinotopic areas V1-V3 by means of a phase-encoded retinotopic mapping approach. A fast Fourier transformation was applied to each voxel’s time series to identify activation that corresponded to the frequencies of the wedge and ring stimuli. The phase lags (i.e., the resulting polar angle and eccentricity maps) were then overlaid onto the reconstructed, inflated cortical surface (obtained via FreeSurfer, Martinos Center for Biomedical Imaging, Boston, MA, USA). We then defined the borders of V1–V3 as reversals in the polar angle map. The resulting masks served as regions-of-interest (ROIs) in further analyses.

### Classification

#### Image Statistics Classifier

For assessing how much information about the material property in question was contained in the image statistics, we applied a classifier to the image statistics. For each observation (i.e., image), we built a feature vector (i.e., *z-*scored image statistical parameters). We then trained the classifier on our set of images and tried to predict for each image if it belonged to the high- or low-ratings group, i.e., if it scored high on the property in question or low. Note that we did this for each property separately, thus we always conducted a two-way classification between images with high and low property ratings, for example “smooth” vs. “rough.” Since we built the groups from the same pool of 84 material images, several of the images appeared in more than one group. All classification analyses were performed using code that employed the linear discriminant analysis implemented in the classify function of the statistics toolbox for MATLAB (versions R2012a and R2013a^3^). The function was used with the option ‘diaglinear’ which fits a multivariate normal density to each group and estimates the covariance matrix based on the diagonal. Out of interest, we also conducted the classification analyses with a Support Vector Machine (SVM) implemented in MATLAB (Statistics Toolbox). This yielded very similar results to the ones obtained with the discriminant analysis. Therefore, in this manuscript, we will focus only on the results of the discriminant analysis.

#### Multivoxel Pattern Analyses (MVPA)

Multivoxel pattern analyses, in contrast to ‘traditional’ fMRI analysis methods, allows for the analysis of how much information about a certain feature is contained in a particular brain area, even if this feature does not lead to an average activation difference, for example because the neurons coding for two different conditions are intermingled. In MVPA, a classifier is applied to the voxel patterns just as we did with our sets of image statistics.

For decoding property ratings from brain activity patterns, we first set up a general linear model (GLM) in SPM for each participant. In the GLM, we included a separate regressor for each of the 84 stimuli. We then masked the resulting β*-*maps (one for each image) with our ROIs. The voxel values within each ROI were vectorized and used as feature vectors in the classification procedure. Again, we built a two-way classifier for each of our material properties to tell apart images with high and low ratings.

#### Leave-One-Out Cross-Validation and Permutation Tests

Classification performance accuracy can be greatly overestimated when a classifier is tested on the same dataset it is trained on. Therefore, we employed a leave-one-out cross-validation. In this procedure, the classifier is iteratively trained on all but one observation and then tested on the remaining observation. Specifically, each classifier was run 42 times, each image serving once as test observation. This ensures that the training and test sets remain independent throughout the classification procedure.

One-tailed *t-*tests were used to compare the mean accuracies across subjects to chance performance (50%). To test the significance of the image statistics classifier and the MVPA in a stricter manner, we additionally used a bootstrapping procedure. We ran each classifier repeatedly, each time permuting the observations’ group labels in a random fashion. From these permuted classifications we estimated our classifiers’ chance distributions.

#### Searchlight Analysis

In order to exhaustively search the brain for material property information, we applied an exploratory searchlight analysis in addition to the ROI-analyses (Kriegeskorte et al., 2006). In this analysis, a spherical searchlight is placed at all possible locations in the brain. Then, an MVPA was conducted within each searchlight sphere in order to explore where in the brain information about material properties is contained. For this, we masked the β-maps of each participant with the participant’s gray-matter mask. On each voxel in turn, we centered a searchlight in the form of a sphere with a 4 voxel radius (e.g., Bannert and Bartels, 2013; de Haas et al., 2013). This searchlight acted as a mask. We built a feature vector out of the voxel values within the mask and employed a leave-one-out classifier to distinguish between high- and low-rating images. For each searchlight, the classifier’s performance accuracy was written back to the center voxel. Consequently, for each participant and material property, we obtained a searchlight accuracy map. From these, we subtracted the classifiers chance level (50%), and combined individual participants’ accuracy maps in a second-level analysis in SPM to detect group effects.

## Results

### Image Ratings

**FIGURE 2:**
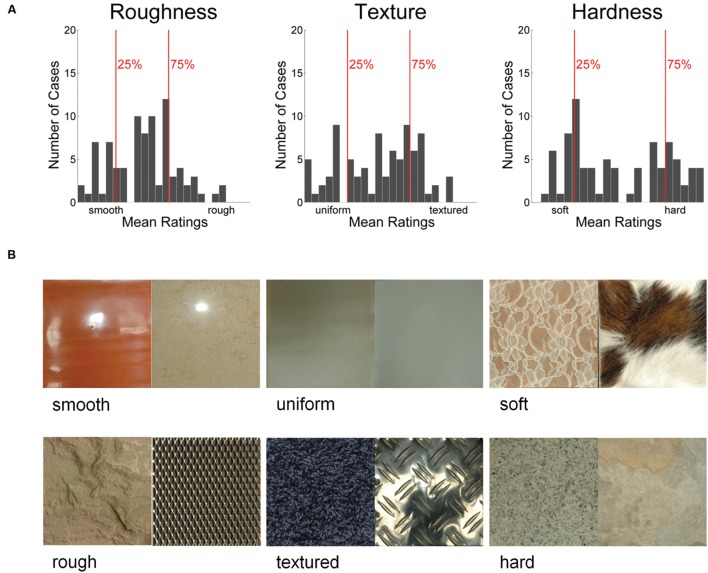
**Rating distributions (average ratings of six observers) for all 84 images for each of the three material properties **(A)** and example images **(B)**.** Vertical red lines indicate the 25th and 75th percentile of ratings. Images with ratings below the 25th percentile made up the low-rating images for that property, and images above the 75th percentile made up the high-rating images. The cutoffs were at 2.3 and 4.2 for roughness, at 2.5 and 4.7 for texture, and at 2.5 and 5.7 for hardness.

The high- and low-rating groups were based on participants’ ratings. **Figure 2** shows the distribution of ratings for each property.

**FIGURE 3:**
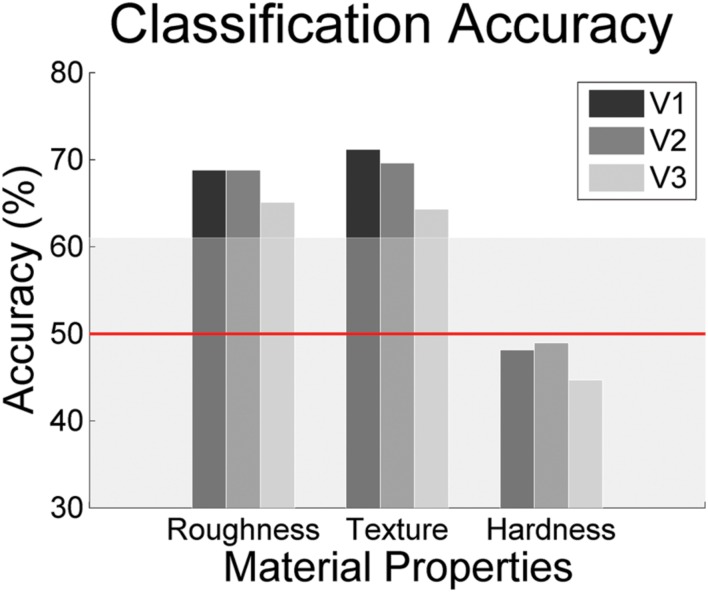
**Classification accuracies of a discriminant analysis in retinotopically defined visual areas V1-V3 (nine participants).** Again, the horizontal line indicates chance level (0.5). The shaded area indicates the *p*-threshold resulting from the permutation test (*p* < 0.05, one-tailed test). The threshold was averaged across all participants.

In order to estimate the amount of information about the material property groups contained in our participants’ brain activity patterns while they observed our material stimuli, we ran the classifier on the β-weights extracted from areas V1, V2, and V3 in the subset of participants who had completed the retinotopy (**Figure 3**). The pattern of results is rather similar to that found with visually responsive voxels, with best classification performances for roughness and texture.

### Image Statistics

**FIGURE 4:**
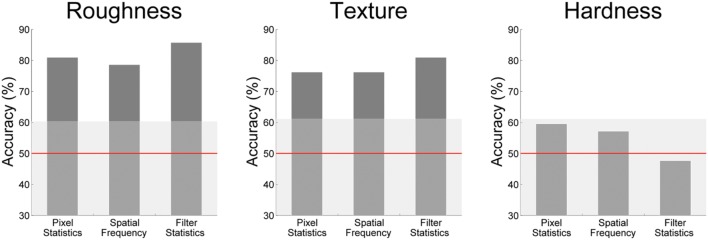
**Accuracies of the image statistic classifier.** The shaded area indicates the *p*-threshold resulting from the permutation test (*p* < 0.05, one-tailed test, 500 permutations).

To see how much information about our image groups was contained in the different sets of image statistics, we applied a classifier to the statistics derived from the Portilla and Simoncelli algorithm. **Figure 4** shows the results of the image statistics classifier. Results are reported for the following groups of image statistics: (1) marginal statistics of the pixelwise luminance distribution, (2) spatial frequency parameters, (3) filter parameters. All Portilla and Simoncelli statistics performed rather well in the classification of high and low roughness and texturedness. Interestingly, even the ‘simple’ spatial frequency and pixel statistics lead also to relatively good classifications; they outperform the Portilla and Simoncelli filter and pixel statistics.

Roughness could be classified well by the Portilla and Simoncelli parameters, which of course capture important low-level aspects (e.g., contrast) as well as crucial texture appearance (e.g., granularity, lack of directionality). However, spatial frequency information performs even better in predicting high and low roughness.

For texturedness, all image statistics perform similarly as for roughness, indicating that similar features can be used to discriminate high and low texturedness and roughness. This might stem from the fact that even though roughness and texturedness are different concepts, at least the images that were rated as low in both properties are very similar, namely smooth, textureless surfaces.

Hardness was not classified above chance. This makes sense because hardness *per se* does not have a proper visual correlate. Instead, higher level factors, like category perception and learning come into play in the visual perception of hardness.

**FIGURE 5:**
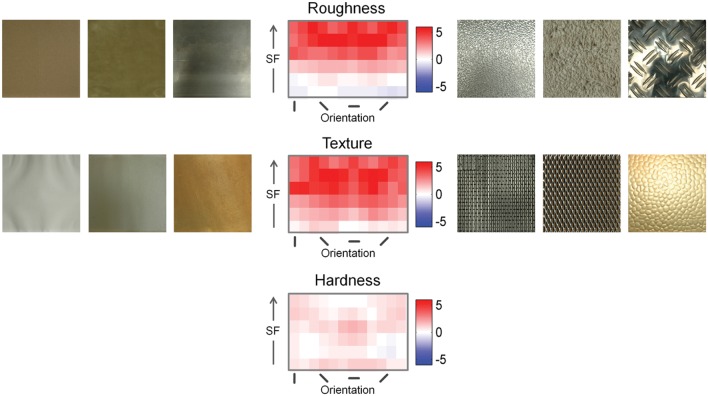
**Comparisons of steerable pyramid subbands between images with high and low ratings.** The steerable pyramid is a bank of multi-scale, multi-orientation band-pass filters, spanning roughly one octave in bandwidth at each scale. Spatial frequency increases from bottom to top. Spatial frequency increases from bottom to top, ranging approximately from 0.07 to 4.6 cpd in logarithmic steps. Orientation represents the orientation of components in the Fourier spectrum. Positive *t*-values mean that the high-rating images have more energy in that subband. Left and right of the graph are example images that were correctly classified into the low- and high-rating groups, respectively. As examples we chose those images with the highest probabilities of belonging to their respective group as estimated by the classifier. Since the classifier performed at chance for images with high and low hardness rating, no material images are depicted here.

Seeing that spatial frequency alone performed quite well for visual material properties, we wanted to explore which oriented spatial frequency subbands discriminate best between images with high and low ratings. We therefore conducted *t*-tests to compare high- and low-rating groups with regard to the energy contained in each subband of the steerable pyramid. The results of this analysis are shown in **Figure 5.** Clearly, for roughness and texture, mid- to high-frequency bins contain more energy than low-frequency bins.

For surfaces to appear rough, they need to have higher power at medium and high spatial frequencies, while the appearance of texturedness requires energy at the low to medium spatial frequencies. Except from very low spatial frequencies, however, images with high levels of both properties show higher power in almost all frequencies except very low ones. This makes sense considering that for both properties, images that received low ratings were images of smooth, homogeneous surfaces, as shown in the example images.

For hardness, the differences are obviously very small and could therefore not be picked up by our classifier.

### Consistency between MVPA and Image Statistics Classifier

Having shown that we can classify material properties using image statistics and brain activation patterns, the crucial question is of course whether the features we extracted from image analysis are in fact the ones used by the visual cortex. Therefore we compared the consistency between the image statistics classifier and the fMRI classifier to the amount of agreement we would expect from chance based on the accuracies of the two classifiers. Specifically, we checked if the labels that the two classifiers gave each single image (high vs. low) coincided more often than expected under and assumption of independence between the two classifiers. This procedure is similar to and inspired by the choice probability approach (Britten et al., 1996) that was used to study the relationship between cell activity and behavior. It was, for example, also applied by Stone and Krauzlis (2003) to study if motion perception and oculomotor signals are driven by shared neuronal substrates. Both of these approaches look for common variance of two measures in order to determine the degree to which those two measures are connected. We used it because we were interested in the association between image statistics and neural response.

An agreement between image statistics and brain activation emerges, of course, by the fact that both aim to classify the perceptual judgment. To avoid this problem, we looked at the two poles of each property separately. We took, for example, the 21 images judged to show rough surfaces, and calculated how many were classified as rough using Portilla and Simoncelli filter statistics or brain activation, in this case 14 and 16.2 (averaged across 15 participants) images. Based on these numbers, we then computed (for each participant) the consistency we would expect if the two classifiers were independent (averaged across 15 participants: 59%) and compared that to the actual consistency of the two classifiers (averaged across participants: 67%). We did the same analysis for images low-rating images, in that case smooth images. In essence, we compared the actual agreement to the chance level calculated from the classification accuracies. If the agreement is significantly higher than the chance level we computed, this indicates that both could be driven by the same underlying factors.

**FIGURE 6:**
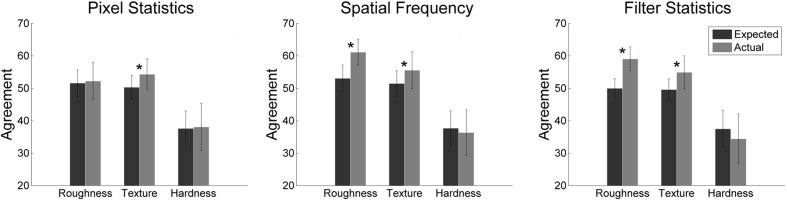
**Summary of the two classifiers’ (image statistics and MVPA) actual vs. expected agreement for the three different image statistic groups.** Error bars indicate standard deviations; asterisks indicate statistical significance (*p* < 0.05).

Summarized matches for each group of image statistics (bars show mean results for high- and low-rating images) are shown in **Figure 6.** The Portilla and Simoncelli filter statistics as well as the spatial frequency statistics show more consistency with the MVPA than we would expect from chance. This indicates that the image statistics are closely associated with the brain activation differences that can be picked up by the MVPA. Both show significant consistencies for roughness and texturedness, but not for hardness. This is, however, expected, since the MVPA performed at chance for hardness. For texturedness, the classifier with pixel statistics shows more overlap with the labels given by the MVPA than we would expect from chance. This suggests that spatial frequency information and filter statistics, but also grayscale pixel statistics partly drive the differential response patterns for high and low rating images in early visual cortex. We analyzed all pixel statistics separately and found that minimum and maximum contribute most to classification.

### Searchlight Analysis

**FIGURE 7:**
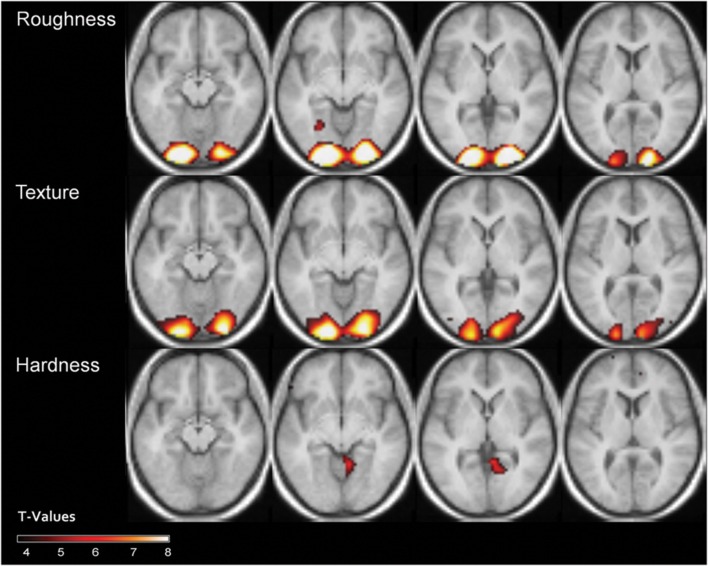
**Results of the whole-head searchlight classification procedure for the different properties [*t*(14) = 3.79, *p* < 0.001].** Transverse slices at *z* = 7, 0, -7 and -14.

Since the pattern classification in visually responsive voxels and V1, V2, and V3 showed rather similar results, we wanted to look at where information about material properties is contained in BOLD patterns across the brain in a more exploratory manner. The searchlight results are summarized in **Figure 7** and **Table 1.** The results from the searchlight analysis confirm those of the mask analysis, showing that best discrimination can be achieved for roughness, and texturedness, especially in early visual areas. Beyond early ventral stream, we could not observe significant above chance classification accuracy within the visual system. High and low property ratings could obviously be decoded best from early visual areas. For hardness, we did not find significant classification accuracies in early visual areas. Instead, we found above chance classification accuracy in the right lingual gyrus (see **Table 1** for a complete list of labeling results).

**Table 1:** Overview of the significant clusters (*k* > 5) identified with the searchlight approach.

MNI-Coordinates	Size	Location	Labeling	Probability	*T*-Value
**Roughness**					
-21 -98 -3	2011	Left inferior occipital gyrus	hOC3v (V3v)	60%	19.81
24 -88 -2		Right lingual gyrus	Area 17	30%	17.43
-27 -61 -5	32	Left lingual gyrus	hOC4v (V4)	20%	3.87
**Texture**					
24 -94 -11	1912	Right lingual gyrus	Area 18	60%	9.69
-15 -94 -8		Left inferior occipital gyrus	Area 18	50%	9.40
-39 -82 -2		Left middle occipital gyrus	hOC5 (V5)	10%	4.46
**Hardness**					
9 -55 -2	129	Right lingual gyrus	Area 18	50%	6.35


## Discussion

Our results indicate that information about material properties is to a large degree contained in low-level image statistics, and that these image statistics could be also reflected in brain activity patterns evoked by material images.

### Image Statistics

The image statistics classification analysis showed that high and low levels of roughness and texturedness could very well be decoded with the features of the Portilla and Simoncelli texture model. The model produces impressive synthesis results and seems to contain sets of features that are perceptually important (Balas, 2006). It aims at describing textures in terms of non-spatial summary statistics, and has been shown to mimic filter responses in V1 and the computations carried out with these filter responses at a later stage in the early visual system, presumably in V2 (Freeman and Simoncelli, 2011; Freeman et al., 2013). Therefore it is not surprising that it can capture aspects of material property perception, even though this has not previously been shown.

More surprisingly, roughness and texturedness could be classified even better with spatial frequency parameters. Neumann and Gegenfurtner (2006) could show in their study that spatial frequency statistics were able to predict the perceived similarity of natural images rather well, and Balas (2008) could show that participants’ grouping of natural textures could even better be predicted with a simple frequency power spectrum model than with the Portilla and Simoncelli model. This is clearly in accordance with our results and especially impressive when considering that most of the images in our high- and low-rating groups were not extreme examples of their respective property spectrum. Obviously, spatial frequency features lack complexity in order to exhaustively explain the computations done by the visual system when extracting material information. However, the fact that these features perform rather well indicates that the visual system might rely on them to quite some degree in order to obtain information about material properties.

In recent years, it has been pointed out that the visual system probably relies on sets of invariant image statistics, or cues, in order to estimate object and material properties, instead of carrying out costly computations to work out the physics of a visual scene (for a review, see Fleming, 2014). For example, Ho et al. (2006, 2007) found that the lighting angle of a surface affects perceived roughness. They explained this with a model according to which the lighting angle of the surface affects luminance and contrast, which then leads to a change of the roughness percept, even though participants viewed the stimuli stereoscopically and therefore, had true 3D-information available. Within their model, they identified luminance and roughness cues that observers could use for roughness estimation. They concluded that the visual system could in principle rely on such a simple statistics as contrast to estimate a material’s roughness. Similarly, spatial frequency has been suggested as another candidate cue for material property perception. Giesel and Zaidi (2013) found that the perception of 3D material properties undulation, thickness, and roughness could systematically be altered by increasing or decreasing the energy of specific spatial frequency bands of a material photograph. The exact image-based cues employed by the visual system are not known, but it is clear from the earlier results and from the results presented in this study that the brain can rely on statistics such as the moments of the luminance and color distribution or spatial frequency information. Doing so seems like an efficient heuristic.

Hardness could not be decoded reliably by means of our image statistics. In a previous study, we could show that observers can make accurate hardness judgments based on visual information (Baumgartner et al., 2013). However, there are no simple, straightforward statistics that could explain this. Judging hardness without touch might rely on a combination of perception, memory and cognition. Therefore, the described image statistics are of little help in the decoding of hardness.

### Representation of Material Properties in the Brain

In the present study, we chose to present each stimulus image only once. We did this because of our relatively large pool of images, and because we wanted to ensure the BOLD response to a previous trial to diminish substantially before the beginning of a new trial. Kay et al. (2008) have shown that image identification is possible from single trials. We therefore consider our approach valid. We even consider it strength of the present study that we were able to decode image information from single trials.

The results from the retinotopically defined masks as well as those from the searchlight approach suggest that there is a lot of information about material properties in early visual areas. As the results from the image statistics classification analysis show, very simple image statistics, in particular luminance and spatial frequency information, as well as the filter parameters defined by Portilla and Simoncelli, can be quite informative about certain types of material properties. The involvement of these could be the driving force for the classifier results in early visual areas. This notion is confirmed by the comparison of the labels assigned by the images statistics classifier and the MVPA. The effects of this analysis seem to be moderate, which is, however, expected, taking into account the noisiness of fMRI data and the fact that each material image was seen only once by each participant. All together, our results argue for an involvement of low-level image statistics in material perception. This agrees well Hiramatsu et al. (2011), who found highest accuracies in V1 and V2 for classification of material categories. The few studies that have examined the neural basis of surface/material perception have found that tasks associated with visual material perception lead to an increase in activation in medial regions of the ventral extrastriate cortex, especially the collateral sulcus and parahippocampal gyrus (Newman et al., 2005; Cant and Goodale, 2007, 2011; Cant et al., 2009; Cavina-Pratesi et al., 2010). In contrast, shape perception is known to be associated with lateral parts of the ventral stream (Malach et al., 1995). While these studies have made a substantial contribution to our understanding of cortical processes during the perception of different visual features, it is also quite evident that the task of understanding material property perception is complex and needs to be looked at in detail. Nishio et al. (2012), in a single-cell recording study, could show that perceived level of glossiness, independent of shape, material, or illuminant, was coded by neurons in the inferior temporal lobe—in other words, in a region relatively high up in the visual processing hierarchy. However, in a recent fMRI study, which also investigated gloss perception in monkeys, Okazawa et al. (2012) found higher BOLD signals in the whole ventral stream (from V1 to the inferior temporal lobe) in response to glossy objects (when compared with matte or scrambled objects).

This discrepancy in the results of the two studies demonstrates once again that it is important to not only look at the perceptual consequences of materials but also take image-based information and its propagation through the visual system into account. However, no brain imaging study has so far looked at the differential processing of individual material properties and especially the image-based features that the brain relies on. In the present study we could show a close correspondence between low-level image statistics, brain activity in early visual areas, and perception. Our results indicate that low-level image statistics are reflected in brain activity patterns elicited by images of materials. This confirms and expands Hiramatsu et al.’s (2011) results who cleverly demonstrated that the representation of rendered material surfaces changes from a more image-statistics based one in early visual areas to one that emphasizes perceptual similarity between materials in higher regions of the ventral pathway.

In addition, the high classification accuracies that could be achieved with some of our low-level image statistics suggest that they might contribute directly to the perception of materials. The visual system could in principle rely on these image statistics to get a first idea of the visual input, similar to the “gist” that has been suggested to guide scene processing (Oliva and Schyns, 2000; Torralba and Oliva, 2003). For example, image statistics have been shown to affect the perception of gloss (Motoyoshi et al., 2007; Wiebel et al., 2015), while at the same time they still leave open contributions of higher level aspects of the stimuli (Kim and Anderson, 2010; Kim et al., 2011; Marlow et al., 2011).

The results for our two ‘visual’ properties, roughness and texturedness, are quite similar, both in terms of the image statistics classifier as well as the MVPA classifier. When we trained the MVPA classifier on roughness, it could also decode texturedness above chance, and vice versa. Obviously, at this low level, the two properties cannot be discriminated. Keeping the results of the image statistics classifier in mind, which performed similarly for the two properties, this is not surprising. Obviously, there have to be more elaborate computations that can later judge materials in a more fine-grained manner, carried out in higher visual areas. Such computations could, for example, take 3-dimensional information into account to determine a surface’s roughness, or access haptic representations of a surface (Goda et al., 2016). So, even though low-level statistics, namely pixel statistics, filter parameters and spatial frequency information seem to play a role in material perception, mid- and high-level areas of the visual system must be involved in the perception of material properties. So why did we observe the above-chance classification accuracies in early visual areas? The increasingly complex processing and larger receptive field sizes and therefore blurred retinotopy in areas higher up the ventral stream might render the patterns there less reliable for decoding material properties. Even though Hiramatsu et al. (2011) found the representation of materials to correspond better to the perceived image similarity, they also found highest classification accuracies in earliest areas. In addition, in our case it is rather hard to say what participants actually did in the scanner when watching the materials, therefore the processing higher up in the visual stream might have been subject to some variation between participants and trials. But note that for hardness, the searchlight analysis yielded above chance accuracy in the anterior lingual gyrus. It has been suggested that ventromedial regions of the visual system are crucial to texture and material perception (Cant and Goodale, 2007, 2011; Jacobs et al., 2014), so we consider it likely that this result reflects more complex material-related computations.

### Limitations and Outlook

We are aware that with the present correlative data we cannot ultimately conclude that our image statistics cause the perception of material properties or that the perception of certain material properties is what causes the above-chance classification accuracy. Our data do, however, provide a strong link between simple image statistics and brain activity in response to material images. Low-level aspects of the images contain information that co-varies with judgments of material properties.

There are also a few further limitations of our study that are caused by its exploratory nature. Both the stimulus set and the set of properties are, of course, limited. Our choices were mainly motivated by the idea to link these experiments to our previous research using the same stimuli. We originally chose the property ‘texturedness’ because we aimed for a purely visual property. However, there is a distinction between visual texture and surface texture (Bergen and Landy, 1991) that our participants did not seem to make in their ratings. The overall high correlation between the results for texturedness and roughness suggests this, too. This is probably also caused by our limited stimulus set that did not contain many textured, i.e., patterned surfaces.

Our stimulus set is also limited with respect to the flat mounting of our materials. We chose flat stimuli on purpose because we wanted to eliminate variance due to shape and restrict ourselves to surface cues. This could be handled differently in future studies.

In addition, the rating task we chose for our participants in the scanner is slightly problematic with respect to the variations it probably caused in our participants. They were instructed to pay attention to the materials and material properties. We did not apply a fixation task because we also wanted to explore possible effects of high-level material processing.

In the future, this work should be pursued with parametrically modulated stimuli in order to overcome the restrictions of our correlative approach and seek evidence for a causal relationship between low level image statistics and material perception.

## Conclusion

For us to perceive materials with all their properties there must be an interaction of various complex computations in the brain. As we could show here, rather simple image statistics and low-level image features contain much information about various material properties and seem to contribute to their neural processing.

## Author Contributions

EB and KG designed the experiment and wrote the manuscript, EB collected and analyzed data.

## Conflict of Interest Statement

The authors declare that the research was conducted in the absence of any commercial or financial relationships that could be construed as a potential conflict of interest.

## Footnotes

1 http://www.allpsych.uni-giessen.de/MID/.

2 http://www.cns.nyu.edu/∼lcv/texture/

3 http://www.mathworks.com
